# Validation of the Brazilian version of the processes of change questionnaire in weight management in adults with overweight and obesity in Brazil

**DOI:** 10.1186/s13098-025-01613-y

**Published:** 2025-07-18

**Authors:** Quênia de Carvalho, Paola Rampelotto Ziani, Bruno Braga Montezano, Jeferson Ferraz Goularte, Adriane R Rosa

**Affiliations:** 1 Graduation Program in Pharmacology and Therapeutics, University Federal of Rio Grande do Sul (UFRGS), Porto Alegre, Brazil; 2 Laboratory of Molecular Psychiatry, Experimental Research Center, Hospital Clinic of Porto Alegre (HCPA), University Federal of Rio Grande do Sul (UFRGS), Porto Alegre, Brazil; 3 Graduation Program in Psychiatry and Behavioral Sciences, University Federal of Rio Grande do Sul (UFRGS), Porto Alegre, Brazil; 4 Laboratory of Molecular Psychiatry, Hospital das Clínicas de Porto Alegre (HCPA), University Federal of Rio Grande do Sul, Avenida Ramiro Barcelos, 2350, Porto Alegre, Zip Code 90035-903 RS Brazil

**Keywords:** P-Weight, S-Weight, Transtheoretical model, Psychometry, Weight loss

## Abstract

**Background:**

In Brazil, 57.5% of men and 62.6% of women are considered overweight, highlighting obesity as a public health issue. Obesity increases the risk of various chronic diseases, and most people struggle to maintain weight loss in the long term. The Transtheoretical Model is an approach that considers readiness for behavioral change, and questionnaires like the Process of Change Questionnaire (P-Weight), and the Stages of Change Questionnaire (S-Weight) have been developed to assess these changes in weight management. This study aims to analyze the adapted version of the P-Weight in Brazil, relate stages and processes of change, and investigate its correlation with external measures related to eating disorders.

**Methods:**

A total of 656 adults participated in the study, including people in weight loss treatment and people from the general community. All participants responded to the P-Weight), the S-Weight, and the Eating Attitudes Test (EAT-26), which assesses the risk of eating disorders used as a measure of external validity. Socio-demographic variables were also investigated.

**Results:**

The 33-item P-weight questionnaire showed satisfactory psychometric properties with high internal consistency (Cronbach’s alpha = 0.959). Exploratory and confirmatory factor analyses revealed a 4-factor model similar to the original Spanish version of P-Weight with a slight rearrangement of the items (KMO = 0.92, df (528, *n* = 328) = 8,401.015; *p* < 0.0001). We found significant associations between processes and stages of change (*p* < 0.001) and a moderate correlation between the four subscales of P-weight and EAT-26 (*p* < 0.001). Finally, the mean score of P-Weight was higher in the clinical sample compared to the general community, suggesting the sensitivity to discriminate cases and controls (*p* < 0.001).

**Conclusion:**

This study showed the validity and reliability of the Brazilian version of the P-Weight scale. Therefore, the P-Weight is readily available to help professionals employ precision interventions for weight loss considering the patient’s motivational stage in combination with their individual use of the cognitive processes of change.

**Supplementary Information:**

The online version contains supplementary material available at 10.1186/s13098-025-01613-y.

## Introduction

In Brazil, the percentage of adult people who are overweight is 57.5% for men and 62.6% for women [1], which suggests that obesity is a public health problem. Current approaches emphasize that lifestyle changes with the adoption of low-energy dietary patterns effectively reduce body weight and may prevent obesity-associated chronic diseases [3]. However, most people with obesity do not stay on weight loss programs long enough, and only a few of them lose and maintain the ‘new’ weight [4]. Indeed, a study (*n* = 50,081) showed that 95% of people who try to lose weight regain it in five years after a dietary, pharmacological, or behavioral intervention; the faster the weight loss, the faster the weight recovery, with this behavior more evident in cases than control groups [5]. Therefore, the long-term success of weight loss programs depends on the ability of individuals with obesity to change their behaviors, which is extremely difficult for most people, especially regarding maintaining a new diet pattern and exercising regularly [6].

Considering behavior as the key to weight loss, The Transtheoretical Model (TTM) proposed by James Prochaska in 1979 has been used in many fields of Medicine, including obesity [7]. According to the TTM, stages of change represent the temporal, motivational, and constancy aspects of behavior modification, focusing on “when” change occurs. These stages include Pre-contemplative (no intent to change), Contemplative (ambivalence about change), Preparation (planning and commitment), Action (implementation of change), and Maintenance (sustaining change over time) [9]. In contrast, processes of change describe “how” individuals engage in activities to modify complex behaviors. Furthermore, processes of change have been identified as predictors of behavior change in interventions aimed at health promotion, such as quitting smoking, healthy eating behavior, physical exercises, and weight control [4].

The identification of stages and processes of change is critical to individualizing interventions and may help to increase the effectiveness of the treatment. For that, specific questionnaires to assess stages and processes of change in weight management have been proposed by the scientific community [12]. The Stages of Change Questionnaire (S-Weight) and Process of Change questionnaire in weight management (P-Weight) seem to be effective in assessing the readiness for change and the processes faced during change in patients who are controlling their weight [13]. Both instruments were validated in populations of Spain [4] and the United Kingdom (UK) [14] with acceptable psychometric properties, which allow their use to implement behavior change interventions for weight loss. When applied to individuals with obesity on the waiting list for bariatric surgery, P-weight and S-weight showed they were in the Action and Maintenance stages for change. There was also a relationship between stages for change and the body mass index (BMI) since the BMI is lower in individuals in the Action and Maintenance stages compared to those who reported being in the Precontemplation stage for change [6]. Therefore, the identification of these stages and processes of change may contribute to weight loss and increased postoperative success rates.

A Thai randomized clinical trial using the P-Weight and S-Weight scales demonstrated that individualized nutrition counseling combined with the TTM model led to greater weight loss in overweight and obese individuals compared to a control group receiving only an educational handbook [15]. Another recent study found that patients with obesity and a recent stroke who are emotionally invested in weight loss and actively address their weight are more likely to be in an active phase of behavior change [16]. Although studies using the P-Weight and S-Weight scales have shown promising results, a Cochrane review reported that the effectiveness of TTM in promoting weight loss is still unclear due to the small sample and heterogeneity in the methodologies used, making it difficult to draw effective conclusions [10].

Thus, considering the high prevalence of overweight and obesity in Brazil and the absence of validated scales capable of assessing stages and processes of changes in individuals with overweight and obesity, the aim of this case-control study was: (1) to analyze the psychometric properties of the transcultural adapted Brazilian version of the P-Weight (Supplementary Material 1); (2) to assess the relationship between stages and process of change; and (3) to assess the relationship between P-Weight and external measures regarding eating disorders such as Eating Attitudes Test (EAT).

## Materials and methods

### Translation, transcultural adaptation, and content validity

Initially, the P-Weight scale was independently translated from Spanish to Portuguese by two bilingual translators (QC and ARR). Then, the back translation was done by a native Brazilian Portuguese (DB) speaker who was unaware of the study objectives. Then, the responsible researchers (QC, JG, and ARR) adapted semantic and idiomatic equivalences. In the next step, the instruments were administered to ten specialists (clinical nutritionists) to detect possible divergences in the understanding of the item by Brazilians. In addition, each item was evaluated in terms of cultural equivalence. All considerations made by specialists were reviewed by our research team. Then, a final Brazilian Portuguese version was obtained and applied to a sample of ten patients undergoing weight loss treatment to verify its applicability.

The P-Weight is composed of 34 items to measure behavior change processes. For each question, on a 5-point Likert scale, the person scores according to their level of agreement for the question. The P-Weight suggests that four processes of change are involved in this setting: (i) emotional re-evaluation (EmR; emotional reactions to being overweight and what will happen if they engage in weight management actions); (ii) weight consequences evaluation (WCE; the individual’s awareness of consequences that overweight has on their life, becoming aware that actually, they have a weight problem); (iii) weight management actions (WMA; those specific actions that people engage when trying to manage their weight); (iv) environmental restructuring (EnR; actions aimed to modify the individual’s environment to promote weight management) [14]. The scores for each of the four change processes are calculated by adding the scores obtained on the items belonging to the same subscale. None of the items are reverse-scored. To make the scores of the different subscales comparable, these scores are transformed into a scale from 0 to 100, with 0 being the non-use of a given process of change and 100 being the full use of such process [14]. The highest use of a process is represented by scores above 50, whereas the lowest use of a process by scores below 50 [15].

### Sample, inclusion criteria, and recruitment

A cross-sectional observational study was carried out using an anonymous online questionnaire. The sample was non-probabilistic by convenience (*n* = 656), composed of people from the general community (community sample) and participants in weight loss programs at the Hospital de Clínicas de Porto Alegre (HCPA) and private clinics in Porto Alegre (clinical sample). The inclusion criteria for the community sample were individuals aged over 18 years and with a BMI above 18.5 kg/m^2^, and the inclusion criteria for the clinical sample were individuals aged over 18 years, with a BMI above 18.5 kg/m^2^, and participation in nutritional or pharmacological treatment for weight loss or waiting for a bariatric surgery. In the clinical sample, pregnant and lactating women were excluded.

The community sample was recruited via an online questionnaire available on social networks, via an access link on the REDCAP platform; and the clinical sample was collected at the HCPA Nutrition and Bariatric Surgery outpatient clinics and private weight loss treatment clinics. In the clinical sample, the research team contacted patients, inviting them to participate in the research, and an individual and exclusive link was generated on the platform for each invitee, which was made available only when the person agreed to participate. To minimize selection bias and to ensure that each participant answered the questionnaire only once—to avoid duplicates—a question was inserted at the end of the consent form: *“- Have you answered this questionnaire before?”* When answering yes, the questionnaire was automatically excluded. At the end of the survey, all participants who wished to, receive, as a form of thanks, an online material on Mindful Eating. The study followed the conditions established in Resolution 466/12 of the Brazilian National Health Council (CNS) and was approved by the HCPA Research Ethics Committee (CAAE: 54352521.8.0000.5327 N. 2021.0447). Before entering, the survey participants were asked to confirm their eligibility and to sign an informed consent form.

To calculate the sample size, a P-Weight questionnaire with 34 items was considered, with a 0.05 margin of error, a 95% confidence level, and a 0.85 expected Cronbach’s alpha [14]. Thus, the total sample was initially estimated at 640 individuals, 320 for the clinical sample and 320 for the community sample.

### Other instruments

#### S-Weight

The S-Weight consists of five mutually exclusive items, among which participants had to choose to be allocated to one of the five stages of change (Pre-contemplative (PC), Contemplative (C), Preparation (P), Action (A), Maintenance (M)), as proposed by the TTM. According to the S-Weight, the Action stage involves efforts to lose weight for less than 6 months, while the Maintenance stage refers to sustaining these changes for over 6 months, indicating habit consolidation [11].

#### Eating attitudes test-26 - (EAT-26)

This scale is used to assess eating disorder risk [17]. In Brazil, the EAT-26 was validated in female adolescents with satisfactory psychometric properties, consists of 26 items, and is divided into three subscales: (1) dieting, (2) bulimia and food preoccupation, and (3) oral control. The scoring is based on a Likert scale, with a cutoff point of ≥ 20 indicating a higher risk for eating disorders [18]. The EAT was used in the UK and Spanish study in adults with obesity [14].

### Data analysis

Descriptive analyses were performed using the PAWS Statistics 18 software, whereas the R software version 4.1.3 was used for exploratory (EFA) and confirmatory (CFA) factor analyses. For the EFA and CFA analyses, the total sample (*n* = 656) and participants from the community and clinic samples were all grouped into the same group and then randomized to obtain two comparable subgroups regarding age (*p* = 0.6) and gender (*p* = 0.81). Following the cross-validation method used in the original article [11, 14], Subgroup 1 (*n* = 328) was used to perform EFA and Subgroup 2 (*n* = 328) for CFA. A cross-validation is desirable for EFA and CFA when the sample is large enough to randomly distribute participants into at least two groups, and it is potentially useful to perform EFA with half the sample and then use CFA to validate the factor structure with the other half [19]. The Oblimin rotation with eigenvalues ​​>1 with the extraction of four factors and degree of item extraction > 0.3 [4] was applied to the EFA for Subgroup 1. Then, the CFA was used on the other half of the sample (Subgroup 2) to confirm the internal structure of the questionnaire. Third, internal consistency was performed for both subgroups using Cronbach’s alpha. Analysis of variance (ANOVA) was applied to the total sample (*n* = 656) to assess the relationship between processes (dependent variables) and stages of change (independent variables) and analyze the trend in the use of processes of change. Spearman’s correlations were used to assess the convergent external validity between the P-Weight and the EAT-26.

## Results

### Participants

A total of 1,018 individuals were initially invited, of which only 656 correctly completed the questionnaire, so the participation rate was 64.44%. Most participants (70%) were from the city of Porto Alegre. The mean age was 41.53 (SD = 11.28) with a BMI of 30.8 kg/m^2^ (SD = 7.10). Table 1 shows other sample characteristics.

**Table 1:** Sociodemographic characteristics of the sample (*n* = 656)

Characteristic	Community (*n* = 330)	Clinical (*n* = 326)	Total (*n* = 656)
**Gender, % Female**	81.50	74.50	78.00
**Age, years (a)**	41.21 (11.46)	41.84 (11.11)	41.53 (11.28)
**BMI (a, b), Kg/m** ^**2**^	28.41 (5.45)	33.24 (7.71)	30.81 (7.10)
**% Educational level**			
Higher education	63.63	70.85	67.22
High School	36.36	29.14	32.77
**% Skin-color**			
White	85.00	83.12	84.29
Non-white	15.00	16.87	15.70
**% Marital status**			
Married	65.45	59.50	62.5
Not married	34.54	40.50	37.5
**% Family income**			
< R$ 7,272.00	66.06	85.27	75.61
> R$ 7,272.00	33.93	14.72	24.39

a = Standard deviations b = Self-reported BMI

### Internal structure

EFA was applied to Subgroup 1 (*n* = 328). The Kaiser–Meyer–Olkin test indicated the sample adequacy for the analysis (KMO = 0.92, df (528, *n* = 328) = 8,401.015; *p* < 0.0001) and Bartlett’s test showed a significant value with X [2] (528) = 11,809.46 and *p* < 0.001, (polycor () function of the package psych of the R programing language version 4.1.3 was used). The communalities ranged from 0.412 to 0.914 and the percentage of variance explained with four factors was 73%. The factor loadings of these items reached values ​​above 0.30, ranging from 0.374 to 1.007, as shown in Table 2.

**Table 2:** Factor loadings of P-Weight items to factors in the exploratory factor analysis (*n* = 328)

item	Question	Communalities	Factor loadings*			
			EmR	AWC	EnR	CE
**4**	I now realize I have a weight problem	0.629	0.525			
**9**	I am worried about gaining more weight	0.699	0.772			
**13**	Losing weight would help me improve my relationships with others	0.777	0.493			
**17**	Being overweight makes me feel bad	0.830	0.618			
**21**	I fell guilt when I overeat	0.563	0.644			
**23**	If I lost weight, I would feel better about myself	0.914	1.007			
**25**	I’m not happy with my current weight	0.756	0.828			
**27**	If I lost weight, I would be happier	0.839	0.833			
**29**	I feel good when I am able to control my eating habits	0.654	0.620			
**31**	When I lose weight, I feel proud of myself	0.767	0.813			
**34**	I am committed to losing weight	0.627	0.468			
**2**	I look for information about the types of food that could help me lose weight	0.412		0.497		
**3**	I try to put food away to avoid nibbling	0.506		0.726		
**6**	I tell myself positive things to avoid overeating	0.595		0.728		
**7**	I try not to have food in sight	0.637		0.685		
**10**	When I really want to eat, I do activities to avoid it	0.651		0.702		
**11**	There are no snack foods in my fridge or cupboards	0.651		0.749		
**14**	I have learned to control my appetite	0.620		0.790		
**15**	I avoid places where people eat a lot	0.724		0.816		
**18**	I have learned skills that reduce my desire to eat (e.g., distracting myself)	0.685		0.705		
**19**	When I am on a diet [1] I avoid eating with people who I overeat with	0.662		0.693		
**22**	I avoid buying high-calorie food	0.585		0.728		
**26**	To avoid overeating I prefer eating at home or cooking my own food	0.754		0.645		
**24**	I am aware that there are more and more people who encourage me to lose weight	0.821			0.682	
**28**	My family and friends praise me for not overeating	0.811			0.566	
**30**	My family and friends congratulate me when I manage to lose weight	0.770			0.529	
**32**	People around me support me in trying to lose weight	0.852			0.723	
**33**	I have someone who listens to me when I need to talk about my being overweight	0.510			0.374	
**5**	Society’s view of obese people affects me emotionally	0.527				0.463
**8**	My weight restricts my relationships	0.818				0.609
**12**	My current weight makes my daily life difficult	0.846				0.554
**16**	My family and friends are worried about my weight	0.879				0.614
**20**	Most of my health problems are due to my being overweight	0.762				0.424
	Eigenvalues		17.55	3.83	1.63	1.13
	Explained variance proportion (%)		53	65	70	73

EmR = emotional re-evaluation; AWC = actions for weight control EnR = environmental restructuring; CE = Consequences of excess weight. *Extraction of factor loadings using function fa () psych package maximum likelihood method (ML), R program version 4.1.3

**Fig. 1:**
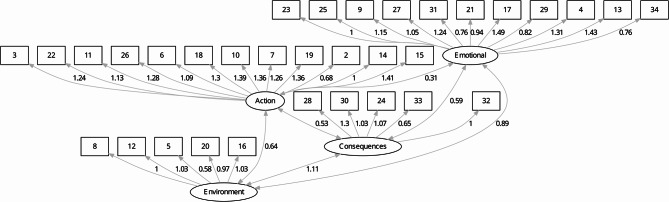
Diagram representing the four first-order factors model for processes of change in weight management. Emotional = EmR = emotional re-evaluation. Action = AWC = actions for weight control. Environment = EnR = environmental restructuring. Consequences = CE = consequences of excess weight

The four factors revealed at EFA were labeled according to their specific content, following the same idea of ​​the original nomenclature given by Andrés 2011: (1) Emotional Re-evaluation (EmR) with 11 items; (2) Actions for weight control (AWC) with 12 items; (3) Consequences of excess weight (CE) with five items; and (4) Environmental Restructuring (EnR) with five items. One item (*“I think I should eat food with less fat”)* was excluded since the degree of extraction was less than 0.3 and the communality was less than 0.2. Therefore, the final Brazilian version of P-weight consists of 33 items, as demonstrated in the Z Material. Figure 1 shows the diagram representing the four process factors of change in weight management.

CFA was performed with Subgroup 2 (*n* = 328), excluding data from Subgroup 1 for this analysis. The measurement model consisted of four factors loaded according to the results found in the EFA, showing an acceptable fit of the 33 items. The SRMR (0.077) falls within the range considered acceptable. The RMSEA (0.083) is close to the range deemed satisfactory. Furthermore, the CFI (0.863) and TLI (0.852) indices, although slightly below the threshold of 0.90, reflect a satisfactory fit of the model, particularly when considering the characteristics of the study. Table 3 presents the CFA structure with unstandardized parameters [20]. The complete table is in the Supplementary Material 1 (library function, lavaan package, R program version 4.3.1).

**Table 3:** Indices obtained in the confirmatory factor analysis

	CFI comparative fit index	NFI/TLI normed fit index /Tucker-Lewis Index	SRMR standardized root mean square residual	RMSEA Root mean square error of approximation
Model 1	**0.863**	**0.852**	**0.077**	**0.083**

### Internal consistency

Internal consistency was assessed for the four subdomains of P-weight derived from internal structure analysis and for the total scale in both Subgroups 1 (sample 1) and 2 (sample 2). Corrected item-total correlations were adequate for all items since all of them reached values over 0.30 (Table 4).

**Table 4:** Internal consistency analysis of the 4 factors P-Weight questionnaire in both subgroups and the total sample

	Number of Items	SAMPLE 1 (*N* = 328)		SAMPLE 2 (*N* = 328)		Total sample (*N* = 656)	
		Cronbach’s alpha	Item-total correlations (range)	Cronbach´s alpha	Item-total correlations (range)	Cronbach´s alpha	Item-total correlations (range)
EmR	11	0.933	0.558–0.827	0.925	0.541–0.803		
AWC	12	0.922	0.480–0.746	0.921	0.477–0.763		
EnR	5	0.884	0.515–0.788	0.871	0.436–0.763		
CE	5	0.904	0.576–0.834	0.900	0.547–0.811		
						0.959	0.377 a 0.776

EmR = emotional re-evaluation. AWC = Actions for weight control. EnR = environmental restructuring. CE = consequences of excess weight

### Relationship between processes and stages of change

**Fig. 2:**
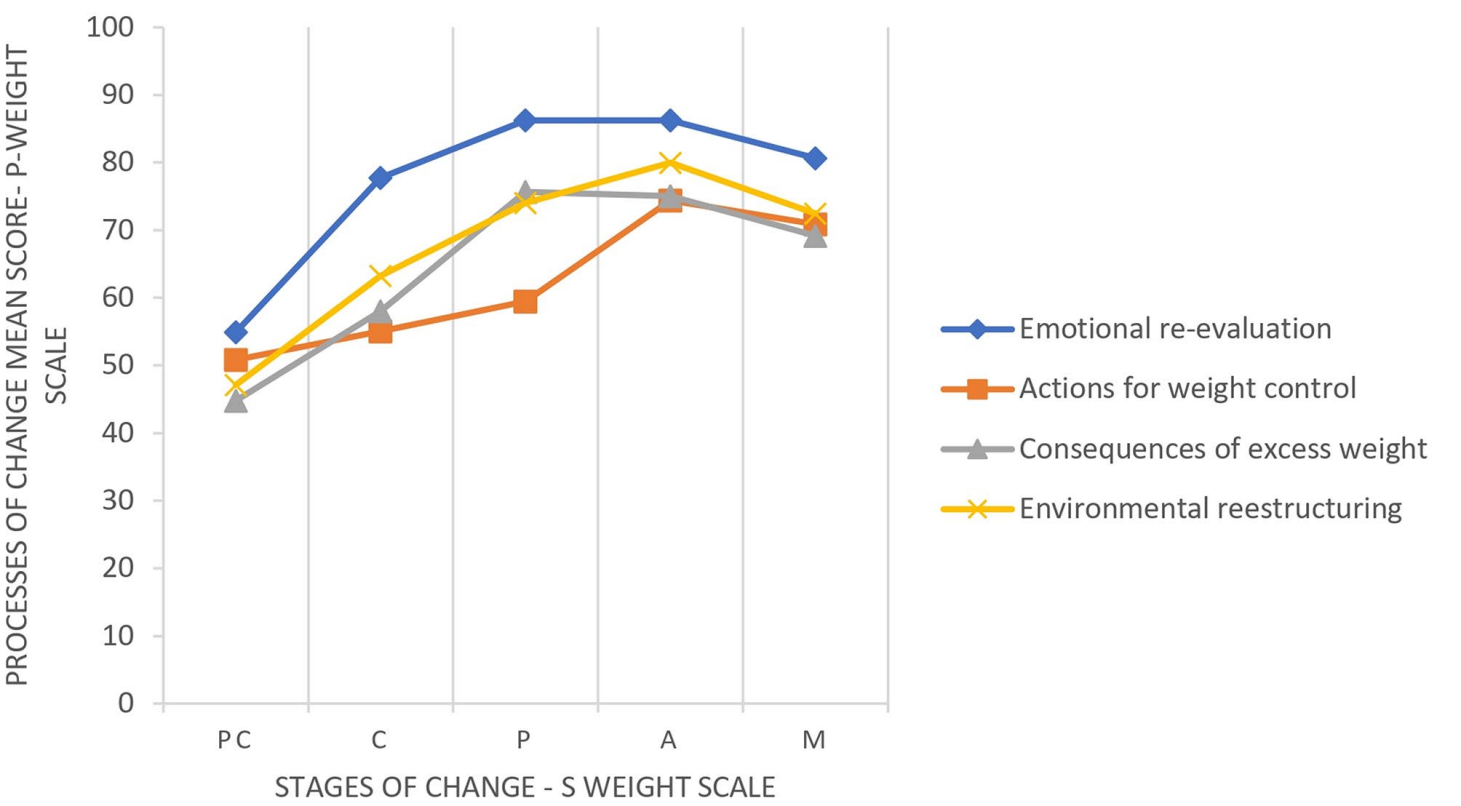
Analysis of processes of change scores across stages of change. PC = pre-contemplation; C = contemplation; *P* = preparation; A = action; M = maintenance

As shown in Fig. 2; Table 5, ANOVA revealed significant differences in processes of change scores across the five stages of change. These differences are illustrated in the very strong linear association, with P-weight subscale scores increasing from PC to A stages of changes, except for the “consequences of excess weight (CE)” that reached the peak in the P stage. Regarding the S-weight, most participants (223) were in the Action stage, which suggests that most of them were making efforts to manage their weight. From these, 155 participants (23.6%) were from the clinical group, whereas 68 (10.4%) participants were from the community group.

**Table 5:** Analysis of variance of processes of change scores across stages of change (ANOVA)

	PC mean (SD)	C mean (SD)	*P* mean (SD)	A mean (SD)	M mean (SD)	F*(Sig)
EmR	54.92 (17.02)	77.76 (12.85)	86.24 (9.86)	86.24 (12.43)	80.67 (14.93)	60.55 (0.000)
AWC	50.83 (14.15)	55.03 (14.91)	59.48 (14.73)	74.40 (19.47)	70.87 (17.14)	40.24 (0.000)
CE	44.80 (18.23)	58.00 (19.76)	75.66 (22.32)	75.04 (23.92)	69.15 (24.94)	25.05 (0.000)
EnR	47.12 (18.00)	63.25 (16.78)	79.98 (19.15)	74.43 (21.26)	72.44 (21.35)	36.20 (0.000)
Clinical sample (n) (%)	**1 (0.15)**	**7 (1.06)**	**42 (6.40)**	**155 (23.6)**	**121 (18.4)**	**326 (49.7)**
Community sample (n) (%)	**49 (7.47)**	**95 (14.5)**	**42 (6.40)**	**68 (10.4)**	**76 (11.6)**	**330 (50.3)**
Total (n)	**50**	**102**	**84**	**223**	**197**	**656**

PC = pre-contemplation; C = contemplation; *P* = preparation; A = action; M = maintenance; EmR = emotional re-evaluation; AWC = actions for weight control; EnR = environmental restructuring; CE = consequences of excess weight

### Relationship of processes of change with other variables

As shown in Table 6, the subdomains EmR, EnR, AWC, and CE of the P-Weight were significantly correlated with EAT-26, suggesting the convergent validity of the P-weight (*p* < 0,0001).

**Table 6:** Spearman’s correlations between P-Weight and EAT-26

	EmR	AWC	EnR	CE
EAT 26* – Correlation Coefficient	0.579	0.593	0.584	0.638
**p (Sig)**	< 0.001	< 0.001	< 0.001	< 0.001

EmR = emotional re-evaluation. AWC = actions for weight control. EnR = environmental restructuring. CE = consequences of excess weight. *EAT-26 = Eating attitude test-26; validated for the Brazilian population (FORTES et al., 2016)

### Sensitivity

**Fig. 3:**
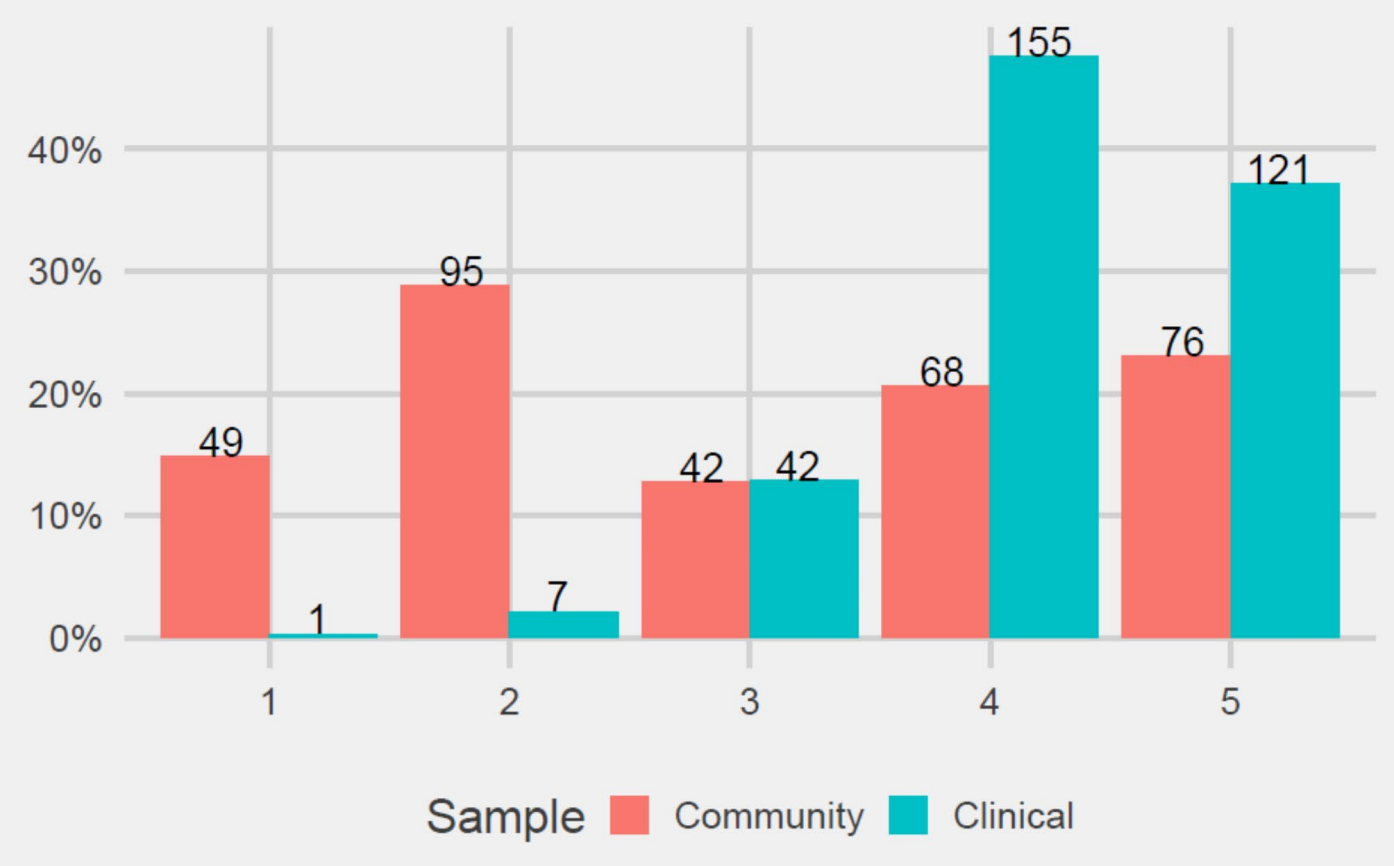
Analysis of stages of change in clinical and community samples. 1 = pre-contemplation (PC) 2 = contemplation (C) 3 = preparation (P) 4 = action (A) 5 = maintenance (M). Y axis = S-Weight mean scores

Mean differences between clinical and community samples were calculated to assess whether the P-Weight and S-Weight were sensitive enough to detect differences between these groups. Results showed that statistically significant differences were obtained in all subscales, with participants in the clinical sample obtaining higher scores. Finally, participants from the clinical sample were allocated to more advanced stages of change for weight management. Table 7; Fig. 3 show it.

**Table 7:** Difference mean scores of the P-Weight subscales between the community and clinical sample

*P*-Weight domains	Community sample (*n* = 330)	Clinical sample (*n* = 326)	Overall sample (*n* = 656)
**EmR**			
Mean (SD)	73.60 (16.4)	88.20 (10.7)	80.90 (15.7)
Median [Min, Max]	75.40 [23.1, 100]	89.20 [43.1, 100]	83.10 [23.1, 100]
**AWC**			
Mean (SD)	56.40 (14.7)	76.90 (17.5)	66.60 (19.1)
Median [Min, Max]	56.70 [20.0, 93.3]	76.70 [28.3, 100]	65.00 [20.0, 100]
**EnR**			
Mean (SD)	58.40 (17.6)	86.70 (14.3)	72.40 (21.4)
Median [Min, Max]	60.00 [20.0, 100]	88.00 [36.0, 100]	76.00 [20.0, 100]
**CE**			
Mean (SD)	53.60 (19.9)	83.40 (19.5)	68.40 (24.7)
Median [Min, Max]	52.00 [20.0, 100]	88.00 [20.0, 100]	72.00 [20.0, 100]

EmR = emotional re-evaluation. AWC = actions for weight control. EnR = environmental restructuring CE = consequences of excess weight

## Discussion

The Brazilian version of the P-Weight showed satisfactory psychometric properties with very high internal consistency, a positive relationship between processes and stages of change (S-Weight), and convergent validity, as indicated by a significant correlation with the EAT-26. As expected, the P-Weight mean score was higher in the clinical sample compared to the community sample, suggesting the discriminative validity of the instrument. Furthermore, the P-Weight resulted in a four-dimensional model, similar to the original Spanish version [4, 14], that is, this instrument is fit to measure attitudes and behaviors involved in weight control. Therefore, the Brazilian version of the P-Weight is readily available for use in both research and clinical practice.

Regarding internal structure, the subscales and the total scale showed adequate results, with a Cronbach’s alpha of 0.959 for the total sample, characterizing the reliability of our instrument. These results corroborate the findings in the Spanish and the UK validation studies, which showed Cronbach’s alpha of 0.960 [4] and values ranging from 0.849 to 0.955, respectively [14]. The Brazilian version of the P-Weight has 33 items, one item less than the original version. The item “I think I should eat food with less fat” was excluded since it has a 0.081 factor loading (less than 0.3), as recommended by Floyd and Widaman [21]. We believe that this happened due to different eating patterns between Europe and Latin America. In Brazil, people present a high consumption of fast-assimilated carbohydrates and low consumption of fatty foods, although carbohydrates- and sugar-rich foods are often also fatty foods. However, it is possible that respondents have not been aware of this technical information, which leads to a misunderstanding of this question.

Factorial analysis identified and confirmed that a model of four correlated processes of change was the most appropriate to explain behavior change in weight management. The four following subscales of the P-Weight obtained were *Emotional re-evaluation (EmR)*,* Action for weight control (AWC)*,* Consequences of excess weight (CE)*, and *Environmental restructuring (EnR)*. Compared with the original Spanish version, the number of items in each domain was similar with some exchange of items between domains, which was also observed in the UK validation study [14]. For example, the question “I try to put food away to avoid nibbling” in the original Spanish version is in the Environmental restructuring (EnR) domain, and the UK and Brazilian versions, it was rearranged to the Actions for weight control (AWC) domain. This is justified because it is a specific Action item, which is taken by the individual to lose weight and, at the same time, it is related to the environment in which the individual is inserted. Likewise, the question “I now realize I have a weight problem,” which in the Spanish and the UK versions is included in the Consequences of excess weight (CE) domain, in the Brazilian version, was rearranged to the Emotional Revaluation (EmR) domain; this can be explained because, besides its representation as a consequence, this item also represents the individual’s feeling when they understand that they have a weight problem. Therefore, an item can either be in the EmR or EnR domain or in the CE or AWC domain since they are closely related and focus on the actions that individuals take when trying to lose weight, how they feel in this context, how the environment influences this process, and what they understand about the effects generated by excess weight. Thus, 12 items were rearranged in different domains in comparison to previous works; all of them were assessed by the research group that considered this version satisfactory since the subscales are strongly related to each other.

Confirmatory analysis indicates a good overall fit of the model, with the SRMR (0.077) within the acceptable range and the RMSEA (0.083) close to satisfactory. The CFI (0.863) and TLI (0.852) indices, although below 0.90, demonstrate a satisfactory fit, suggesting that the model adequately describes the processes of weight management, with potential for future refinements.

The relationship between the processes and the stages of change was also a noteworthy finding. We observed a gradual increase in the use of processes of change as individuals moved from precontemplation (PC) to action (A) stages. People who were in the PC stage used fewer processes of change compared to people in later stages. The highest use of processes of change was observed in the action (A) stage before declining in the maintenance (M) stage. These results are in line with Andrés’s study that demonstrated that the use of processes of change was lower in the PC stage compared to subsequent stages [4]. Our results also corroborate the findings of a 12-week randomized controlled trial that concluded that the EnR, CE, and AWC processes are higher in the Action stage compared to the previous stages [15]. Another recent study, using the S-Weight and P-Weight scales, revealed that patients with higher scores in the EmR and AWC subscales are more likely to be in the Action stage for weight loss [22].

These findings suggest that when individuals shift their focus from *trying to lose weight* to *trying to maintain weight*, they invest less effort in processes of change. It is also possible that individuals who are closer to their ideal body weight have improved self-esteem and, therefore, remain in the Precontemplation stages [13]. However, this finding may partly explain the high rates of weight regain observed in long-term studies for obesity.

Furthermore, we found a significant relationship between the four subscales of the P-Weight and measures of concern about thinness through the EAT-26, which supports the convergent validity of the scale. As expected, participants who achieved higher scores on using processes of change also achieved higher scores on external measures. These results are in line with previous findings that showed a correlation between P-Weight and EAT-40 among university students and the general population [4] that participated in a program for weight loss.

The TTM has been used to improve changes in health-related behaviors since the 1980s, including smoking cessation and, more recently, weight management [6, 23]. Thus, interventions designed to encourage behavior change are more effective when tailored to the individual’s stage of change [8]. Measuring an individual’s readiness for change in the weight management setting before undertaking an intervention can increase the effectiveness of weight loss interventions, especially in the long term [24]. Therefore, it is crucial to have reliable and validated measurement tools to assess the processes and stages of behavior change in weight management. The Brazilian version of the P-Weight has shown adequate psychometric properties and can be a useful tool to identify the individual’s behavior change processes to their weight. Personalized interventions that promote specific change processes and encourage progress through the motivational stages can aid in weight management.

This study has some limitations that should be considered. The non-probabilistic sampling restricts the representativeness of the Brazilian population and, consequently, the generalization of the results. Self-reported weight and height are subject to biases, compromising the accuracy of the analyses. Additionally, the clinical sample size may not capture the full diversity of patients at different stages of change or undergoing various types of treatment, and the exclusion of pregnant and lactating women limits the applicability of the instrument to these specific populations. As a cross-sectional study, it was not possible to evaluate changes in behavior processes over time or the stability of scores after interventions. The significant correlation between P-Weight and EAT-26, while supporting the scale’s convergent validity, should not be interpreted as an indicator of mental health issues. Finally, the reliance on online platforms for recruitment may have introduced selection bias, excluding individuals with limited internet access. Despite these limitations, the results reinforce the psychometric adequacy of the P-Weight, making it a promising tool for assessing behavior change processes related to weight management.

## Conclusion

The results of this study show that the Brazilian version of the P-Weight has satisfactory psychometric properties, making it a valuable application for weight loss interventions based on the TTM. The use of motivational strategies is crucial to promote adherence to treatments for overweight and obesity. The availability of validated instruments that allow the evaluation of change processes based on the TTM is a great advance and should contribute enormously to this important public health problem. We highlight that individualizing weight management interventions according to each patient’s motivational stage and processes of change allows health professionals to employ more effective treatments according to each individual’s motivational reality.

## Electronic supplementary material

Below is the link to the electronic supplementary material.

Supplementary Material 1

## Data Availability

No datasets were generated or analysed during the current study.

## Abbreviations

TTM: Transtheoretical model
PC: Pre-contemplative
C: Contemplative
P: Preparation
A: Action
M: Maintenance
S-Weight: Stages of change questionnaire
P-Weight: Process of change questionnaire in weight management
BMI: Body mass index
EAT: Eating attitudes test
EmR: Emotional Re-evaluation
WCE: Weight consequences evaluation
WMA: Weight management actions
EnR: Environmental restructuring
AWC: Actions for weight control
CE: Consequences of excess weight
HCPA: Hospital das clínicas de Porto Alegre
EFA: Exploratory factor analyses
CFA: Confirmatory factor analyses
ANOVA: Analysis of variance
CAPES: Coordination for the improvement of higher education personnel
CFI: Comparative fit index
NFI: Normed fit index
TLI: Tucker-Lewis index
SRMR: Standardized root mean square residual
RMSEA: Root mean square error of approximation
