# Supplementary material for: Validation of the Brazilian version of the processes of change questionnaire in weight management in adults with overweight and obesity in Brazil
Source: Diabetol Metab Syndr. 2025 Jul 18;17:282. doi: 10.1186/s13098-025-01613-y (PMC12275437; doi:10.1186/s13098-025-01613-y)
Supplement: Supplementary file 1 — Supplementary Material 1 [file 13098_2025_1613_MOESM1_ESM.docx]

**Supplementary Material**

Attachment 1.

Table 1. P-Weight version validated in Portuguese

|  | Processos de Mudança na Perda de Peso  (P-Weight) |  |  | | | |
| --- | --- | --- | --- | --- | --- | --- |
| item | Questão | DISCORDO  TOTALMENTE | DISCORDO | NÃO CONCORDO  NEM DISCORDO | CONCORDO | CONCORDO  TOTALMENTE |
| 1 | Agora me dou conta que tenho um problema de sobrepeso |  |  |  |  |  |
| 2 | Me preocupa muito a ideia de seguir engordando mais e mais |  |  |  |  |  |
| 3 | Emagrecer me faria melhorar minhas relações com os demais |  |  |  |  |  |
| 4 | Meu excesso de peso faz com que eu me sinta mal |  |  |  |  |  |
| 5 | Sinto-me culpado (a) quando como em excesso |  |  |  |  |  |
| 6 | Se perdesse peso, me sentiria melhor comigo mesmo (a) |  |  |  |  |  |
| 7 | Não gosto do meu peso atual |  |  |  |  |  |
| 8 | Se perdesse peso, seria mais feliz |  |  |  |  |  |
| 9 | Fico feliz quando sou capaz de me controlar com a comida |  |  |  |  |  |
| 10 | Quando perco peso me sinto muito orgulhoso (a) de mim mesmo (a) |  |  |  |  |  |
| 11 | Me comprometo a perder peso |  |  |  |  |  |
| 12 | Busco informação sobre o tipo de alimentação que pode me ajudar a perder peso |  |  |  |  |  |
| 13 | Guardo os alimentos para evitar beliscar |  |  |  |  |  |
| 14 | Digo-me palavras positivas para evitar comer mais do que devo |  |  |  |  |  |
| 15 | Tento não ter comida a vista |  |  |  |  |  |
| 16 | Quando sinto vontade de comer, faço outras atividades para evitar comer |  |  |  |  |  |
| 17 | Tenho a dispensa e geladeira vazias de alimentos para comer ou beliscar |  |  |  |  |  |
| 18 | Tenho aprendido a controlar meu desejo de comer |  |  |  |  |  |
| 19 | Evito lugares onde as pessoas comem muito |  |  |  |  |  |
| 20 | Tenho aprendido habilidades para reduzir meu apetite (meditação e relaxamento) |  |  |  |  |  |
| 21 | Quando estou de dieta evito comer com pessoas que comem em excesso |  |  |  |  |  |
| 22 | Evito comprar alimentos com alto teor de calorias |  |  |  |  |  |
| 23 | Prefiro comer em casa e cozinhar minha própria comida para evitar comer em excesso |  |  |  |  |  |
| 24 | Me dou conta que há cada vez mais pessoas que me animam para que eu perca peso |  |  |  |  |  |
| 25 | Minha família e amigos me dão os parabéns quando não como em excesso |  |  |  |  |  |
| 26 | Meus familiares e amigos me dão os parabéns quando eu consigo perder peso |  |  |  |  |  |
| 27 | As pessoas à minha volta me apoiam para que eu perca peso |  |  |  |  |  |
| 28 | Tenho alguém que me escuta quando necessito falar do meu excesso de peso |  |  |  |  |  |
| 29 | A visão que a sociedade tem das pessoas obesas me afeta emocionalmente |  |  |  |  |  |
| 30 | Meu peso limita minhas relações com os demais |  |  |  |  |  |
| 31 | Meu peso atual dificulta minhas atividades cotidianas |  |  |  |  |  |
| 32 | Meus familiares e amigos estão preocupados com o meu peso |  |  |  |  |  |
| 33 | A maioria dos meus problemas de saúde se devem ao meu sobrepeso |  |  |  |  |  |

Attachment 2.

Table 2. Indices obtained in the confirmatory factor analysis.

| npar | 72.000 |
| --- | --- |
| fmin | 2.020 |
| chisq | 2650.447 |
| df | 489.000 |
| pvalue | 0.000 |
| Baseline.chisq | 16340.571 |
| Baseline.df | 528.000 |
| Baseline.pvalue | 0.000 |
| cfi | 0,863 |
| tli | 0.852 |
| nnfi | 0.852 |
| pnfi | 0.776 |
| ifi | 0.864 |
| rmsea | 0.082 |
| Rmsea.ci.lower | 0.079 |
| Rmsea.ci.upper | 0.085 |
| Rmsea.pvalue | 0.000 |
| rmr | 0.114 |
| Rmr_nomean | 0.114 |
| srmr | 0.072 |
| Srmr_bentler | 0.072 |
| Srmr_bentler_nomean | 0.072 |
| crmr | 0.074 |
| Crmr_nomean | 0.074 |
| Srmr_mplus | 0.072 |
| Srmr_mplus_nomean | 0.072 |
| gfi | 0.758 |
| agfi | 0.723 |
| pgfi | 0.661 |
| mfi | 0.193 |
| ecvi | 4.260 |

**npar**: Number of Parameters.

**fmin**: Minimum Fit Function.

**chisq**: Chi-Square.

**df**: Degrees of Freedom.

**pvalue**: p-Value.

**Baseline.chisq**: Baseline Chi-Square.

**Baseline.df**: Baseline Degrees of Freedom.

**Baseline.pvalue**: Baseline p-Value.

**cfi**: Comparative Fit Index.

**tli**: Tucker-Lewis Index.

**nnfi**: Non-Normed Fit Index.

**pnfi**: Parsimony Normed Fit Index.

**ifi**: Incremental Fit Index.

**rmsea**: Root Mean Square Error of Approximation.

**Rmsea.ci.lower**: RMSEA Confidence Interval Lower Bound.

**Rmsea.ci.upper**: RMSEA Confidence Interval Upper Bound.

**Rmsea.pvalue**: RMSEA p-Value.

**rmr**: Root Mean Square Residual.

**Rmr_nomean**: Root Mean Square Residual (without means).

**srmr**: Standardized Root Mean Square Residual.

**Srmr_bentler**: SRMR (Bentler's version).

**Srmr_bentler_nomean**: SRMR (Bentler's version without means).

**crmr**: Correlation Root Mean Square Residual.

**Crmr_nomean**: CRMR (without means).

**Srmr_mplus**: SRMR (Mplus version).

**Srmr_mplus_nomean**: SRMR (Mplus version without means).

**gfi**: Goodness of Fit Index.

**agfi**: Adjusted Goodness of Fit Index.

**pgfi**: Parsimony Goodness of Fit Index.

**mfi**: McDonald's Fit Index.

**ecvi**: Expected Cross-Validation Index.
